# A prospective randomized double-blind trial of the efficacy of a bilateral lumbar erector spinae block on the 24h morphine consumption after posterior lumbar inter-body fusion surgery

**DOI:** 10.1186/s13063-019-3541-y

**Published:** 2019-07-17

**Authors:** M. B. Breebaart, D. Van Aken, O. De Fré, L. Sermeus, N. Kamerling, L. de Jong, J. Michielsen, E. Roelant, V. Saldien, B. Versyck

**Affiliations:** 1 Department of Anaesthesia, Antwerp University Hospital, Wilrijkstraat 10, 2650 Edegem, Belgium; 2 Faculty of Medicine and Health Sciences, University of Antwerp, Universiteitsplein 1, 2610 Wilrijk, Belgium; 3 Department of Anaesthesia, AZ Klina, Augustijnslei 100, 2930 Brasschaat, Belgium; 4 Department of Neurosurgery, Antwerp University Hospital, Wilrijkstraat 10, 2650 Edegem, Belgium; 5 Department of Neurosurgery, Klina Hospital Brasschaat, Augustijnslei 100, 2930 Brasschaat, Belgium; 6 Department of Orthopaedics, Antwerp University Hospital, Wilrijkstraat, 10 2650 Edegem, Belgium; 7 Clinical Trial Center (CTC), CRC Antwerp, Antwerp University Hospital, University of Antwerp, Wilrijkstraat 10, 2650 Edegem, Belgium; 8 Department of Anaesthesia, Catharina Ziekenhuis, Michelangelolaan 2, 5623 EJ Eindhoven, Netherlands

**Keywords:** Erector spinae block, Lumbar inter-body fusion, Regional anesthesia, Postoperative pain

## Abstract

**Background:**

Spine surgery is associated with considerable postoperative pain and can be challenging to treat. A loco-regional technique suitable for spine surgery should cover the dorsal root of the spinal nerves at the levels where surgery is performed. The erector spinae block is a loco-regional technique with promising results and was recently described at the thoracic level. There are no randomized trials of this technique on a lumbar level. This study tests the hypothesis that the 24-h postoperative morphine consumption is significantly lower in patients undergoing posterior lumbar inter-body fusion surgery with a lumbar erector spinae (LUMBES) block when compared with a sham block.

**Methods:**

This prospective randomized double-blind multicenter study will randomly allocate 80 adult patients undergoing elective posterior lumbar inter-body fusion surgery during general anesthesia to one of two groups as follows: (1) bilateral erector spinae block (20 mL 0.25% levobupivacaine) or (2) bilateral sham block (20 mL NaCl 0.9%). Our primary endpoint is 24-h postoperative morphine consumption. Secondary endpoints include 72-h morphine consumption, intraoperative sufentanil dosage, postoperative pain scores at regular time intervals both at rest and during movement, time to first postoperative mobilization, and the Quality of Recovery 40 survey score.

**Discussion:**

The LUMBES trial is a pragmatic clinical study that will provide evidence of whether a bilateral lumbar erector spinae block is effective in reducing 24-h postoperative morphine consumption in patients undergoing lumbar inter-body fusion surgery. If this hypothesis is confirmed, this finding could contribute to more widespread implementation of this technique.

**Trial registration:**

Local ethics committee B300201837508, ClinicalTrials.gov identifier: NCT03825198. Registered on 31 Jan 2019.

**Electronic supplementary material:**

The online version of this article (10.1186/s13063-019-3541-y) contains supplementary material, which is available to authorized users.

## Background

### Background information

Patients undergoing spine surgery often fear postoperative pain, which can be a source of considerable preoperative distress. In many of these patients, so-called chronic pain, requiring high doses of narcotics and other analgesics, has already been diagnosed. In spine surgery, postoperative pain can often be severe and difficult to treat, certainly if a one-dimensional approach is used to achieve pain control [1, 2]. Many caregivers are reluctant to prescribe liberal doses of opioids to achieve adequate analgesia as this may be associated with side effects such as respiratory depression, sedation, and nausea. Many techniques have been combined in order to decrease opioid consumption after spinal surgery (e.g., epidural catheters, spinal and epidural morphine, or local infiltration) [3]. The introduction of ultrasound has allowed the performance of plane blocks and other techniques such as root blocks and facet infiltrations without the use of unreliable “pop-techniques” or x-ray.

**Fig. 1:**
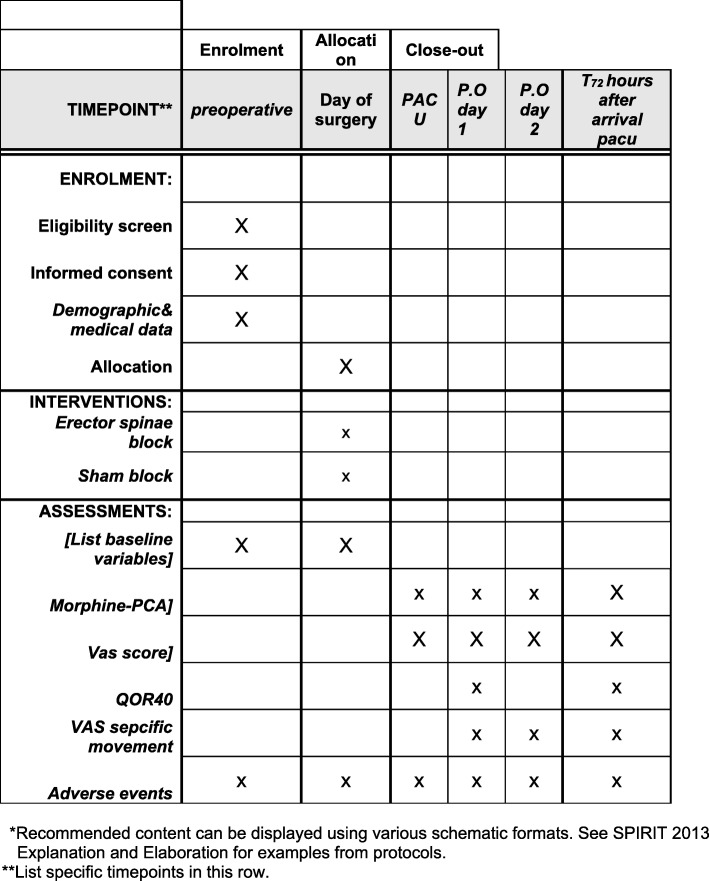
Recommended content for the schedule of enrollment, interventions, and assessments*

A loco-regional technique suitable for back surgery should cover the innervation of the relevant vertebrae and paravertebral muscles and include the dorsal roots of the spinal nerves at this level [4]. Dorsal ramus blocks have been shown to be feasible in the treatment of chronic back pain [5].

Recently, a series of case reports has been described in which bilateral block of the lumbar dorsal ramus nerve resulted in a positive effect on pain scores and morphine consumption after spine surgery [6]. However, to the best of our knowledge, no randomized controlled trials have yet been performed to study the effect on pain scores and opioid consumption. Furthermore, there are promising results for postoperative analgesia with a new plane block, the erector spinae block (ESB), which has recently been described as a safe and simple technique for neuropathic and acute postoperative pain at the thoracic level [7, 8].

### Rationale

Theoretically, an infiltration between the erector spinae muscle and the transverse process provides anesthesia of the dorsal ramus at the same vertebral level. Since the local anesthetic is injected into a plane, the solution can spread both caudally and cranially via the thoracolumbar fascia, resulting in anesthesia of the dorsal ramus of the spinal nerves above and below the injected level. The ESB has been described at the thoracic level with promising results. We performed a small feasibility trial in which we found that the ESB could easily be performed without major inconvenience for the patients (ClinicalTrials.gov identifier: NCT0321453). In this proposed trial, we aim to determine the effect of a lumbar ESB on pain after back surgery, expressed as morphine consumption during the first 24 h postoperatively.

### Objectives and purpose

In this prospective, randomized, double-blind placebo-controlled clinical trial, we will investigate the effect of bilateral ESB (20 mL of levobupivacaine 0.25%) on 24-h postoperative morphine consumption when compared with a sham block (20 mL of NaCl 0.9%) following posterior lumbar inter-body fusion surgery. Secondary objectives include the effect of the ESB on the numeric rating scale (NRS) pain scores in rest at fixed time points: at the time of inclusion, in the post-anesthesia care unit (PACU) (T0 = arrival in PACU, T + 15 min, T + 30 min), and on the ward (twice daily: morning and evening until postoperative day 3). Other secondary objectives are the effect of an ESB on the NRS pain scores during defined movement (mobilization to chair) at 24 h, 48 h, and 72 h; time to first mobilization to chair (hours); time to first walk of 20 m (hours); the required sufentanil dose during surgery (micrograms), total morphine consumption during the first 72 h postoperatively (milligrams), and the Quality of Recovery 40 survey (QoR-40) score at days 1 and 3.

## Methods/Design

### Study design and registration

This is an investigator-initiated prospective randomized double-blind multicenter trial. The study is being performed in accordance with the Declaration of Helsinki (Fortaleza, Brazil; October 2013) and Good Clinical Practice (GCP) guidelines. The study has been approved by the ethics committee at Antwerp University Hospital, Wilrijk, Belgium, and AZ Klina Hospital, Brasschaat, Belgium (reference: B300201837508). The trial has been prospectively registered at https://www.clinicaltrials.gov (reference: NCT03825198) and will be monitored by the clinical trial center of Antwerp University Hospital. Figure 1 displays the schedule of enrollment, interventions and assessments.

### Participation

Patients scheduled for elective 1 or 2 level posterior lumbar inter-body fusion surgery in AZ Klina Hospital and Antwerp University Hospital will be asked for informed consent by a member of the anesthesiology department. Recruitment will occur during the preoperative consultation; it opened on July 9 , 2019, and will close when the required number of patients has been reached.

Inclusion criteria are as follows: (1) American Society of Anesthesiologists (ASA) physical status of 1–3, (2) age of 18–75 years, and (3) normal liver and renal function.

Exclusion criteria are as follows: (1) body mass index (BMI) of less than 20 or BMI of more than 35; (2) allergy to one or more medications used in the study, including epinephrine, levobupivacaine, dexamethasone, propofol, sufentanil, rocuronium, ketorolac, morphine, ketamine, dehydrobenzperidol, ondansetron, and alizapride; (3) chronic strong opioid use (>3 administrations per week); (4) contraindications to a regional anesthetic technique; and (5) patient refusal or no informed consent or both.

### Randomization

Patients will be assigned consecutive numbers upon inclusion in the study. These numbers are randomly allocated (1:1) to the ESB or the sham group by using a web-based randomization system, QMinim. QMinim uses stratified randomization, and stratification will be carried out according to site, gender, and levels of surgery. In QMinim, a minimization procedure is used to randomly assign the patients to ensure a similar distribution of the stratifying arms. Online randomization will be carried out by an independent anesthetist who will also prepare the medication.

### Medication

The ESB study medication will be 20 mL of levobupivacaine 0.25% (Chirocaine, AbbVie, Lake Bluff, IL, USA). The sham group received 20 mL of NaCl 0.9% (B. Braun, Melsungen, Germany).

### Blinding

All investigators, staff, and patients will be blinded to the treatment groups. The study medication will be prepared by an anesthesiologist who is not involved in the study or in the care of the patient. Both solutions and syringes will appear identical. Unless medically indicated, unmasking will occur only after statistical analysis has been completed.

### Interventional treatment

All patients will receive a bilateral ESB. The blocks will be performed by experts in the field of ultrasound-guided regional anesthesia. The blocks will be performed preoperatively in a separate block room with ultrasound after obtaining intravenous (IV) access and application of standard ASA monitoring. The blocks will be placed as described by Chin et al. [6] and modified for the lumbar level. The patient will be placed in the lateral or sitting position. A curved array probe or a high-frequency linear probe, depending on the BMI of the patient, will be placed in longitudinal alignment, 2–3 cm lateral to the vertebral column. The transverse processes of the vertebrae at the level of surgery, the erector spinae muscle, and the psoas muscle will be identified. In case of two-level surgery, the transverse process of the upper level will be considered the target. A 5- or 8-cm 22-G ultrasound needle will be inserted with an in-plane technique in a cephalad-to-caudal direction until bone contact with the top of the transverse process is reached. After slight retraction of the needle, 20 mL of the study medication will be injected behind the erector spinae muscle. The same procedure will be repeated on the contralateral side.

Then general anesthesia—propofol 2–3 mg/kg, sufentanil 15 μg, and rocuronium 0.5 mg/kg—will be induced in a standardized way. After tracheal intubation, anesthesia will be maintained with sevoflurane and intraoperative analgesia provided with sufentanil. The dosages of these agents will be determined at the discretion of the attending anesthesiologist. At the end of surgery, patients will receive acetaminophen 1 g IV, ketorolac 0.5 mg/kg IV (maximum of 30 mg), and a morphine loading dose (0.1 mg/kg) IV to manage postoperative pain. Patients will be extubated in the operating theatre and admitted to the PACU. Postoperative nausea and vomiting prophylaxis will be administered with dexamethasone 5 mg IV just before induction of general anesthesia. This will be supplemented, if necessary, by ondansetron 4 mg IV and further with alizapride 50 mg IV as rescue.

Postoperative pain in the PACU and on the ward will be treated with regular doses of acetaminophen 1 g IV around the clock (four times daily) and by a patient-controlled intravenous analgesia (PCIA) pump containing morphine at a concentration of 1 mg/mL and dehydrobenzperidol 0.05 mg/mL. The PCIA pump will be programmed as follows: no continuous infusion, a bolus dose of 1.5-mg morphine, a lockout interval of 8 min, and an hourly limit of 7.5 mg. If pain management on the PACU is inadequate, defined as an NRS pain score of more than 3 (0 [no pain] to 10 [worst imaginable pain]), additional boluses of 1-mg morphine IV will be administered by the PACU nurses with a total additional dose of morphine limited to 0.15 mg/kg. If pain management with morphine remains inadequate, an IV ketamine (Ketalar, Pfizer, New York, NY, USA) bolus (0.2 mg/kg) will be administered.

### Primary endpoint

The primary endpoint is the morphine consumption during the first 24 h postoperatively in milligrams and will be determined from the PCIA pump.

### Secondary endpoints

As secondary endpoint, the total morphine consumption in milligrams, during the first 72 postoperative hours, will be extracted out of the PCIA pump. Pain scores at rest will be assessed with the NRS score (0 = no pain, 10 = worst imaginable pain) and tested at regular time intervals: at the time of inclusion, in the PACU (T0 = arrival in PACU, T + 15 min, T + 30 min), and on the ward (twice daily: morning and evening until postoperative day 3). Pain scores during defined movement (first moving to a chair and sitting upright) will be registered. Time to first mobilization to a chair (in hours since T0) and time to first walk of 20 m (in hours since T0) will be noted in the patients’ study diary. The QoR-40 score will be calculated from the responses to a standard questionnaire at postoperative days 1 and 3. The QoR-40 is a widely used and extensively validated measure of quality of recovery. It is a 40-item questionnaire on quality of recovery from anesthesia that has been shown to measure health status after surgery [9, 10].

### Tertiary endpoints

Other endpoints include preoperative expected NRS pain score, postoperative nausea and vomiting score according to hospital protocol, number of administered postoperative anti-emetics, time to first meal, and time to first defecation. All block complications or adverse events will be registered.

### Summary of known and potential risks

The ESB is a plane block where a substantial dose of local anesthetic is used. As this technique has only recently been described, limited evidence regarding the potential risks of the block is available. The potential risks described below relate to the known risks of a plane block, facet infiltration, and intramuscular injection:

- Discomfort during puncture
- Allergy for the disinfectant or levobupivacaine (very rare 1:10,000–1:100,000)
- Infection at the skin, needle trajectory, or point of injection (very rare). The clinical presentation can be variable (e.g., redness at the puncture site or in extreme cases an intramuscular abscess). Therefore, the ESB will be performed under strict sterile conditions with a sterile gown, gloves and mask, and a sterile field.
- Bleeding: very rare with the use of an ultrasound-guided technique. When bleeding occurs, this will be noted by the surgeon.
- Neural damage: very rare since the target of the puncture is a muscular plane and not the nerve root or nerve ramus itself
- Local anesthetic systemic toxicity: since the doses are substantial, there is a clinically significant risk for local anesthetic systemic toxicity, as with any existing plane block. It can immediately be treated with intralipid. For this reason, the patient will be monitored during and after the placement of the ESB until the start of surgery. Intralipid should be available in any medical environment where regional anesthesia is performed.

### Data collection

Patients’ demographic data will be collected at the inclusion assessment (height, weight, age, sex, and ASA classification). The attending anesthesiologist will collect data with regard to the anesthesia and surgical procedure. Nurses will collect the data in the PACU. When the patient is transferred to the orthopedic ward, the Acute Pain Service Team will score the QoR-40 on postoperative days 1 and 3, adjust analgesia when necessary, and systematically screen for side effects. Ward nurses will assess NRS pain scores in the morning and evening on postoperative days 1–3. Morphine patient-controlled analgesia (PCA) consumption will be electronically registered by the PCA pump; all other data will be registered by nurses of the PACU, ward pain department, or trial nurses. All medication can be retrieved from the patient data management systems. Complications will be assessed on the day of discharge. During the 72 h of the trial, data will be registered on paper. After termination of the trial (72 h after surgery), the data will be directly registered in the software program Open Clinica.

An independent trial monitor from the Clinical Trial Center at Antwerp University Hospital will conduct a follow-up on the GCP performance of the trial in both study locations. All data will be published anonymously.

### Sample size

Our sample size calculation is based on data from a randomized controlled trial comparing the effect of systemic infused lidocaine with placebo on the 24-h morphine requirement in posterior lumbar arthrodesis [11]. We considered a 25% reduction in PCA morphine consumption to be clinically relevant. To calculate the sample size, we assumed a mean of 51-mg morphine with standard deviation of 19 mg (mean morphine consumption for the placebo group of the above-mentioned trial), a type 1 failure risk of 5%, and a type 2 failure risk of 20%. Thirty-five patients are required in each group to detect a 25% reduction in morphine equivalent over 24 h. The sample size calculation was based on an independent samples *t* test. We plan to include 80 patients in total to compensate for potential dropouts and uncertainty in predicting the actual standard deviation.

### Patient characteristics and baseline comparisons

Demographic and other baseline characteristics will be summarized by treatment group. For categorical variables, frequencies and percentages will be reported. Where values are missing, percentages will be calculated for the available cases, and the denominator will be mentioned. Continuous variables will be summarized as mean with standard deviation or median with interquartile range as appropriate.

Comparisons of demographic and baseline characteristics between the treatment groups will be conducted to assess the effectiveness of randomization. For categorical variables, the chi-squared test or Fisher exact test (when numbers are low) will be used. For continuous variables, a *t* test or Mann–Whitney *U* test will be used as appropriate. The following baseline information prior to randomization will be collected: age, sex, BMI, ASA physical status, indication for surgery, preoperative pain (NRS score), and use of analgesics.

### Analysis of the endpoints

SPSS software version 21 (SPSS, Chicago, IL, USA) or 3.3.2 will be used for statistical analysis. The primary endpoint will be analyzed by using an independent samples *t* test intention-to-treat population (in case of normality).

To evaluate the sensitivity of the results of the primary outcome analysis, a linear regression will be used to model the cumulative morphine consumption during the first 24 h after surgery with treatment as predictor and taking into account possible confounders.

A linear mixed model will be used to model the cumulative morphine consumption over time with subject as a random effect. This model allows correction for confounders and adding a random intercept for site. From this model, the difference in morphine consumption at the different time points can be estimated.

To compare the continuous outcomes (intraoperative sufentanil dosage, required morphine dose, pain scores, QoR-40 score, nausea and vomiting score, and number of administered postoperative anti-emetics) at different time points, we will use an independent samples *t* test if they are normally distributed or a Mann–Whitney *U* test if otherwise. We will also fit a linear regression model for these outcomes, which makes it possible to correct for confounders. A linear mixed model will be studied for the continuous outcomes measured over time.

The time to the different events of interest (first mobilization to a chair, first walk of 20 m, first meal, and first defecation) will be studied in a time-to-event analysis comparing the two treatment arms. We will use a Cox proportional hazard model to adjust for other variables if necessary.

### Dissemination policy

The trial results will be submitted to a peer-reviewed journal regardless of the outcome.

## Discussion

Posterior spine surgery ranks among the most painful surgical procedures and can be challenging to treat. High doses of opioids are often prescribed [1, 2]. Musculoskeletal postoperative pain in posterior approach spine surgery arises from iatrogenic mechanical damage, intraoperative retraction, partial devascularization, and denervation of bone, ligaments, muscles, intervertebral disks, and zygapophysial joints. In addition, neuropathic pain arises from compression and damage to nerve roots exiting the spinal canal and sometimes damage to the spinal cord itself [12]. In order to reduce opioid use, loco-regional and local anesthesia were introduced. In spine surgery, loco-regional techniques were limited to epidural catheters and spinal and epidural morphine. These techniques have side effects and are not routinely used. Local anesthetic wound infiltration is often performed with unfortunately short-lived effect [3]. The loco-regional technique used in this type of surgery should aim to anaesthetize the dorsal root of the spinal nerves at the appropriate operative level [4]. Dorsal ramus blocks have been shown to be feasible in the treatment of chronic pain [5]. In a recent series of case reports, a bilateral block of the lumbar dorsal ramus nerve showed improved pain scores and reduced morphine consumption after spine surgery [6]. Also, there are promising results with the ESB, which has recently been described as a safe and simple technique for neuropathic and acute postoperative pain at the thoracic level [8]. Furthermore, ESB has been shown to effectively control postoperative pain in patients undergoing breast surgery. However, no comparative data for the lumbar level are available [13].

This study will provide clinical evidence on the efficacy of the lumbar ESB in reducing postoperative opioid consumption for posterior lumbar inter-body fusion surgery. If the lumbar erector spinae block (LUMBES) trial demonstrates efficacy, the findings will provide high-quality evidence to support the implementation of this technique in clinical practice. Furthermore, it might trigger studies from other researchers to test our outcomes in their practice.

Potential benefits of the lumbar ESB include the ease of performance with clear landmarks for ultrasound anatomy. The technique is inherently safe, as the target site for injection is a muscular plane and there is practically no risk for mechanical nerve contact. Other benefits include the possible reduction in perioperative opioid consumption. The ESB is performed in patients under anticoagulant therapy or with coagulopathies [8]. Furthermore, hemodynamic instability due to sympathetic blockade, as with epidural and spinal anesthesia, occurs rarely.

Possible risks consist primarily of local anesthetic systemic toxicity. Since substantial doses are considered necessary, there is a clinically significant risk for local anesthetic systemic toxicity, as with any high-volume fascial block. For this reason, patients need to be monitored according to American Society of Regional Anesthesia guidelines with Intralipid available at all times [14].

### Trial status

This document is based on version 8 (Feb. 2, 2019) of the original protocol. We anticipate randomly assigning the first patient on July 9, 2019, and plan to complete the study in February 2020.

## Additional file

Additional file 1: SPIRIT (Standard Protocol Items: Recommendations for Interventional Trials) 2013 Checklist: Recommended items to address in a clinical trial protocol and related documents*. (DOC 125 kb)

## Data Availability

The datasets used or analyzed (or both) during this study are available from the corresponding author on reasonable request and will be accessible only to personnel involved in the trial.

## Abbreviations

ASA: American Society of Anesthesiologists
BMI: Body mass index
ESB: Erector spinae block
GCP: Good Clinical Practice
IV: Intravenous
NRS: Numeric Rating Scale
PACU: Post-anesthesia care unit
PCA: Patient-controlled analgesia
PCIA: Patient-controlled intravenous analgesia
QoR-40: Quality of Recovery 40 survey
